# Strychnia as a Poison

**Published:** 1863-06

**Authors:** Thos. D. Mitchell

**Affiliations:** Professor of Materia Medica and Therapeutics, in Jefferson Medical College, Philadelphia


### STRYCHNIA AS A POISON.

By THOS. D. MITCHELL, M. D., Professor of Materia Medica and Therapeutics, in Jefferson Medical College, Philadelphia.

But a few years ago, no antidote for the poisonous action of strychnia was known, the treatment being purely remedial, and in no sense, chemical. The spasms or jerks were often attempted to be controlled by what we usually style, anti-spasmodics, and such articles were passed into the stomach as are called demulcents, emollients and the like. As a matter of course, the patients generally died, after a brief period of terrible suffering.

In later years, the use of this poison has very greatly increased, partly because of the smallness of the dose, and partly because of the easy methods of concealing its administration. The multiplication of cases, however, has led to a more perfect understanding of its action, and the means of controlling its fatal tendency have had a corresponding increase, so that now we have abundant facilities for meeting the worst cases.

It not unfrequently happens that an individual who has attempted self-destruction by this agency, very soon after the poisonous symptoms develope themselves, announce the reality of his condition, so that the poison being certainly known, we have no difficulty in combatting it. In other cases, no such information can be had, and then we must rely on those marked, prominent signs present, which no practiced eye can ever mistake. The *tetanic jerks or spasms* speaking for themselves, need no interpreter. The physician who is rightly informed understands all this, and decides on instant and vigorous action. He empties the stomach at once, by repeated use of the pump, or by means of a prompt emetic, as of ten grains of sulphate of zinc or sulphate of copper, every ten minutes, until the organ is thoroughly evacuated.

As to the query, "how much strychnia will kill an adult," no fixed answer can be given. Very much depends on the fullness or emptiness of the stomach at the time of swallowing the dose, not a little likewise is due to the previous habits of the patient, the morbid or healthful state of the system, &c. But when a physician is at the bedside of one who is actually under the influence of the poison, after evacuating the stomach as fully as may be, he must lose not a moment in administering the antidote.

The following facts are recited in my lecture on strychnia, at every session, and are now presented to the public in a group, for the purpose of furnishing the profession at large, with an array of means that will be found entirely adequate to any emergency.

Tannic acid and iodine were, for a time, almost the only proper antidotes in use. Both have succeeded, and are therefore reliable. *Braithwaite's Retrospect, part* 42, *page* 311, has evidence in point. The acid may be given dissolved in water, *ad libitum*; at least an ounce should be put in a quart of water, to be drank freely and largely. The use of it forms an insoluble and inert tannate of strychnia.

The tincture of iodine has also proved decidedly antidotal. Give twenty drops in mucilage of gum arabic or sugared water, at once, and in ten minutes after, thirty drops, and, if need be, forty drops for the next dose. This administration controls the spasms, and the patient is safe. An insoluble and inert hydriodate of strychnia is formed in this instance. See *Braithwaite, part* 41, *page* 62.

The *Vermont Caledonian*, July, 1857, says that ninety grains of strychnia were swallowed by a man, in half a pint of strong gin, without his knowledge that the poison was present. As soon as the discovery was made, an emetic was resorted to, and recovery ensued. In this case, we have a manifest instance of the antagonism of poison to poison. The gin alone was competent to kill, and no one can doubt as to the potency of such a mammoth dose of strychnia, *per se*.

A case not very unlike the above is also given. A man who was perfectly drunk under the use of rum, swallowed sixty grains of strychnia at a dose. He recovered. In this instance, as in the other, the alcoholic spirit and the strychnia were antagonistic poisons, either alone having abundant power to kill. Ordinarily, one grain of the alkaloid would destroy life, if there existed no morbid coudition to counteract it.

Camphor has also been found to have an antidotal power; how, in a strict chemical sense, is not, perhaps, well understood. Dr. Claiborne, of Petersburgh, Virginia, reports the case of a man aged thirty, who took two grains of strychnia. In forty minutes he was seen to be laboring under severe jerks or spasms, which continued nearly two hours, almost incessantly. Respiration and deglutition were nearly impracticable. Very large doses of camphor were exhibited, amounting altogether to 60 grains in less than an hour. Recovery ensued.

*Sulphate of morphia* is another antidote, and, of course, opium would prove so. In the *Western Lancet*, Dr. Phillips gives the case of a lady who was poisoned by swallowing three grains of strychnia at a dose, in mistake for sulphate of morphia, which she had long used for a spasmodic affection, and the dose of which had been gradually augmented. On making the discovery, the lady was placed in a very warm bath, and in less than two hours, she was made to swallow five grains of the morphia salt. The action of the poison was completely arrested and she recovered.

*Chloroform* was resorted to by Dr. Jewett, of Boston, (see *Boston M. and S. Journal*) in a boy aged 15, who in mistake swallowed two grains of strychnia. Medical aid was not procured until half an hour after the accident, when the jerks were violent and deglutition almost impracticable. He was relieved by the inhalation of chloroform, for ten minutes, and partial anæsthesia kept up for four hours saved him.

The case reported by Dr. O'Reilly, of St. Louis, is too well known to be detailed here. He saved a patient fully poisoned by strychnia, by the exhibition of table spoonful doses of tobacco. The following experiments, reported in the Dublin *Hospital Gazette*, Dec. 8, 1856, aro in point: Two baths were made, each having five ounces of water, one of them five grains of strychnia, the other five grains of pure nicotina (a most terrible poison and the proximate principle of tobacco). In one of the baths, a frog lived four minutes. A similar frog put in the other, lived one minute. The two baths were then mixed, so that the water now held the strychnia and nicotina in solution. A frog, in all respects like the others, was put into the mixed bath and appeared to be very little injured at the end of 47 minutes, and it did not die till 24 hours had elapsed. The antagonism of the strychnia and nicotina is so obvious, that we need not stop to speak of it.

Still more recently we have an account of the antidotal power of *Hydrocyanic Acid* in the *Medical Times and Gazette* of August 6, 1859. We remark, in passing, that this acid is more speedily fatal than strychnia.

A physician owned a favorite dog, now become mangy and so offensive, that it was decided to kill him with strychnia. An ample portion was given to the beast, but it only set up terrible jerks, without speedily killing, as was anticipated. To relieve the dog from his torture, a drachm of strong hydrocyanic acid was given in a saucer of milk. The whole was lapped up speedily, and soon the animal vomited, got on his legs, ran off a considerable distance and recovered. Here was a most obvious antagonism.

The last antidote to be named is *Arsenious Acid.* On the next day after my lecture on this subject, thre years ago, Surgeon Judson, of the U. S. Navy, handed me a printed slip, taken from *Bell's Life in Sydney*, which shows conclusively, that so terrible a poison as arsenic can control the poisonous action of strychnia. A farmer's grounds were much infested with crows, and to get rid of the pest, he shot an opossum, cut into its body and placed in the cavities a large quantity of strychnia. The opossum thus prepared was hung to the fork of a tree. A favorite sheep dog, attracted by the stranger in the tree, made out, by vigorous efforts, to grasp it, and then to eat freely of the meat. Very soon, he was thrown into tetanic jerks of great severity. The owner resolved to put a period to the animal's suffering by the use of arsenic, a large spoonful blended with water was passed down the throat. Presently the dog was evidently more quiet; the jerks soon ceased, and in one hour recovery was complete.

In this brief paper we have no less than ten articles, each of which is capable of counteracting the poisonous action of Strdchnía, viz: Gin, Rum, Tannin, Iodine, Sulphate of Morphia, Chloroform, Tobacco, Hydrocyanic Acid, Camphor and Arsenic.

Purposely, we have passed over the *modus operandi*, as well as the tests of strychnia, partly because these are of less practical moment to the profession at large, than the immediate treatment of cases; and also because those points have been, as we think, fully met by the wide publication of the celebrated *Palmer* case (in London), and by the numerous essays growing out of that affair. Our main design was to furnish practitioners with such a birds-eye view of the reliable means for the arrest of the poisonous action of strychnia, as can be foond in no volume known to the profession.

Before we dismiss this interesting subject, it may be well to group the points involved in the question, "how much of any poison is competent to destroy life?" This is the more important in view of the obvious lack of information just here.

The points that cross our path in attempting a direct answer to the question cited are:

1st. The purity or worthlessness of the article. Ten drops of Croton oil, we are told, did not seriously hurt a child ten years old, although given at one dose. The oil, however, was very largely adulterated with another oil, and so made harmless. So too, spoiled digitalis leaves, or leaves from a plant raised in soil unfriendly to its perfection, are inert in any dose. Extract of belladonna, utterly decomposed by excessive heat employed in its preparation, would hurt no one in drachm doses.

2d. The condition of the stomach, as to fulness or emptiness. Two men, of the same age and vigor took each an ounce of laudanum on the same day. Both had medical aid in two hours after the accident. The one died, while the other speedily recovered. The full stomach of the one and the empty stomach of the other, accounted for the difference. The one took the poison an hour before the usual dinner time, the other, an hour after he had dined.

3d. The presence in the system, either in the body or mind, of a potent counter-agent, calculated to antagonise the poisonous dose.

The antidotes, named above, for a poisonous dose of strychnia, are in point. The strychnia and the antidote were mutual antagonistics. So too, the case reported in a foreign journal many years ago, of a medical student who, in a fit of desperation amounting to insanity, swallowed twenty grains of acetate of morphia. The terrible mental excitement of the man absolutely controlled the agency of the mammoth opiate dose, and he was restored although not visited until two hours had elapsed. The presence of a full dose of liquid chloride of soda in the stomach of the *Fire King* or American Buffoon as he was called, saved the man from the poisonous action of a drachm of hydrocyanic acid swallowed in the presence of hundreds of wondering spectators, and it is on the very same principle that alcoholic spirit taken until complete intoxication results, is a well known expedient to save life after the bite of the most venomons serpent. The bane and the antidote are perfect antagonists. While, therefore, one grain of any known poison might kill an adult in full health and with an empty stomach, another person of the same age might swallow, with comparative impunity, ten or twenty grains of the same poison, under circumstances such as those above stated.

1009 Clinton St., May, 1863.—*Canada Lancet.*

---

☞ Business letters to the JOURNAL should be addressed to Dr. E. INGALS, Chicago, Ills., P. O. Drawer 5787. When money is received a receipt will be returned with the next number of the JOURNAL, and subscribers failing to get such receipt will confer a favor by giving us notice of the omission.

